# NF-κB Hyper-Activation by HTLV-1 Tax Induces Cellular Senescence, but Can Be Alleviated by the Viral Anti-Sense Protein HBZ

**DOI:** 10.1371/journal.ppat.1002025

**Published:** 2011-04-28

**Authors:** Huijun Zhi, Liangpeng Yang, Yu-Liang Kuo, Yik-Khuan Ho, Hsiu-Ming Shih, Chou-Zen Giam

**Affiliations:** 1 Department of Microbiology and Immunology, Uniformed Services University of the Health Sciences, Bethesda, Maryland, United States of America; 2 Institute of Biomedical Sciences, Academia Sinica, Taipei, Taiwan; Fred Hutchinson Cancer Research Center, United States of America

## Abstract

Activation of I-κB kinases (IKKs) and NF-κB by the human T lymphotropic virus type 1 (HTLV-1) trans-activator/oncoprotein, Tax, is thought to promote cell proliferation and transformation. Paradoxically, expression of Tax in most cells leads to drastic up-regulation of cyclin-dependent kinase inhibitors, p21^CIP1/WAF1^ and p27^KIP1^, which cause p53-/pRb-independent cellular senescence. Here we demonstrate that p21^CIP1/WAF1^-/p27^KIP1^-mediated senescence constitutes a checkpoint against IKK/NF-κB hyper-activation. Senescence induced by Tax in HeLa cells is attenuated by mutations in Tax that reduce IKK/NF-κB activation and prevented by blocking NF-κB using a degradation-resistant mutant of I-κBα despite constitutive IKK activation. Small hairpin RNA-mediated knockdown indicates that RelA induces this senescence program by acting upstream of the anaphase promoting complex and RelB to stabilize p27^KIP1^ protein and p21^CIP1/WAF1^ mRNA respectively. Finally, we show that down-regulation of NF-κB by the HTLV-1 anti-sense protein, HBZ, delay or prevent the onset of Tax-induced senescence. We propose that the balance between Tax and HBZ expression determines the outcome of HTLV-1 infection. Robust HTLV-1 replication and elevated Tax expression drive IKK/NF-κB hyper-activation and trigger senescence. HBZ, however, modulates Tax-mediated viral replication and NF-κB activation, thus allowing HTLV-1-infected cells to proliferate, persist, and evolve. Finally, inactivation of the senescence checkpoint can facilitate persistent NF-κB activation and leukemogenesis.

## Author Summary

Transcription factors of the NF-κB/Rel family are critical for the proliferation of lymphocytes and the expression of genes that mediate inflammatory and immune responses. They are often aberrantly activated in human cancers, especially leukemia, where they confer survival and proliferation advantages. Through the study of the trans-activator/oncoprotein, Tax, of the human T-lymphotropic virus type 1 (HTLV-1), we have found that persistent and potentially oncogenic activation of NF-κB triggers a defense mechanism that commits cells into senescence, an irreversible state of cell cycle arrest. This checkpoint is turned on by hyper-activated p65/RelA and is mediated by two cyclin-dependent kinase inhibitors, p21 and p27, in a p53- and pRb-independent manner. It is often impaired in cancer cells with constitutively active NF-κB. Our results anticipate that the anti-sense protein of HTLV-1, HBZ, which down-regulates NF-κB and HTLV-1 trans-activation by Tax, would mitigate or prevent Tax-induced senescence. This prediction has been borne out experimentally. Thus, Tax promotes robust HTLV-1 replication, potent NF-κB activation and senescence, while HBZ attenuates Tax-driven viral replication and NF-κB activation to allow proliferation of infected cells and persistent infection. Finally, our data support the notion that inactivation of the senescence checkpoint facilitates chronic NF-κB hyper-activation, a critical step in leukemia development.

## Introduction

Human T-lymphotropic virus type 1 (HTLV-1) is the causative agent of adult T-cell leukemia/lymphoma (ATL), a malignancy of CD4+ T cells that develops in 2–5% of infected individuals over a course of several decades [1], [2]. HTLV-1 transactivator/oncoprotein, Tax, is thought to play a key role in ATL development. Tax is a potent activator of HTLV-1 transcription and NF-κB [2]. To promote viral mRNA synthesis, it acts as an adaptor that links three 21-bp Tax-responsive enhancer elements in the HTLV-1 long terminal repeats (LTRs) to transcription factors CREB/ATF-1 and transcriptional co-activators CBP/p300, p300-CBP-associated factor (PCAF) and transducers of regulated CREB (TORCs) [3]–[5]. Activation of NF-κB by Tax is mediated by direct binding of Tax to the regulatory subunit of I-κB kinase (IKK), NF-κB essential modulator (NEMO), also known as IKKγ. This interaction results in constitutive activation of IKKα and IKKβ, degradation of all I-κBs, and activation of both classical and alternative NF-κB pathways [6]–[8]. The oncogenic activity of Tax in cell culture and in transgenic mice is driven by NF-κB [9]–[13].

We have shown recently that Tax can activate the anaphase promoting complex/cyclosome (APC/C), an E3 ubiquitin ligase that controls metaphase to anaphase transition and mitotic exit [14]. APC/C activation by Tax leads to premature poly-ubiquitination and degradation of mitotic regulators including cyclin B and Skp2 [14]. Skp2 is the substrate-recognition subunit of another E3 ubiquitin ligase known as SCF^Skp2^, which mediates the destruction of cyclin E/A-CDK2 inhibitor (CKI), p27^Kip1^ (referred to as p27 henceforth) during S/G_2_ [15], [16]. The premature degradation of Skp2 as induced by Tax leads to SCF^Skp2^ inactivation and p27 protein stabilization. The mRNA level of another CKI: p21^CIP1/WAF1^ (referred to as p21 henceforth) also increases dramatically as a result of promoter activation and mRNA stabilization brought on by Tax [17]. The massive surge in p21 and p27 in turn leads to p53- and pRb-independent cellular senescence termed Tax-induced rapid senescence (Tax-IRS) [14].

Here we show that the p21-/27-mediated cellular senescence induced by Tax constitutes a cellular checkpoint response towards chronic NF-κB hyper-activation. We further demonstrate that RelA is the primary effector of this pathway and RelA acts upstream of the APC/C and RelB to stabilize p27 protein and p21 mRNA respectively. Our results suggest that Impairment to the p21-/27-mediated senescence checkpoint facilitates constitutive NF-κB activation and leukemia development. This is best evidenced by the loss of p27 expression and cytoplasmic localization of p21 in HTLV-1-transformed T cells [14], [18]. Importantly, we found that the HTLV-1 anti-sense RNA-encoded bZip protein, HBZ, could mitigate Tax-induced senescence by virtue of its ability to inhibit NF-κB [19]. HBZ has been shown previously to block *trans*-activation of viral mRNA expression by Tax [20], [21]. Thus, active HTLV-1 replication characterized by potent Tax-mediated LTR and NF-κB activation leads to senescence, while moderation of Tax activities and attenuation of viral replication by HBZ allow HTLV-1-infected T cells to proliferate, expand, and persist.

## Results

### NF-κB activation by Tax correlates with senescence induction

**Figure 1:**
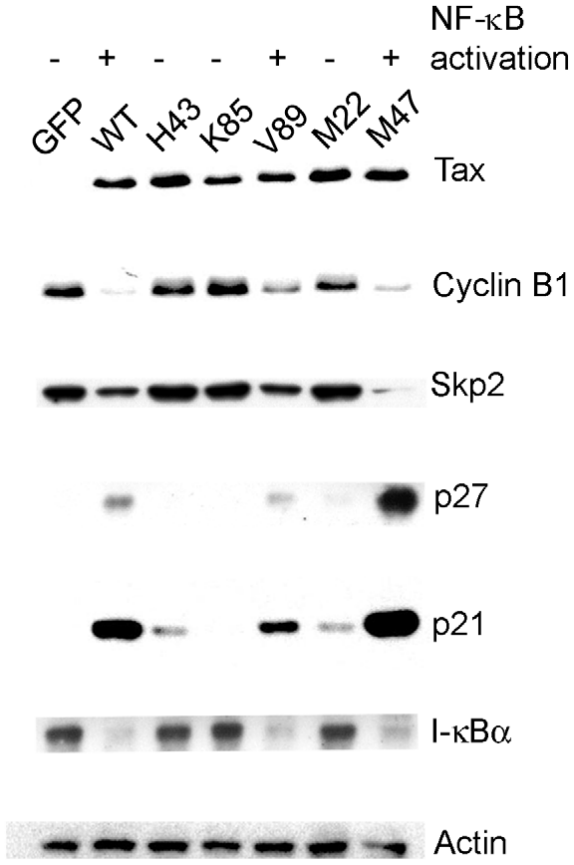
NF-κB activation by Tax correlates with induction of cellular senescence. HeLa cells were transduced with lentivirus vectors carrying the EGFP (GFP) gene and wild-type and mutant *tax* alleles (H43Q, V85A, V89A, M22 [T130A, L131S], and M47 [L319R, L320S]) respectively at an MOI of 5, grown for 72 hours, and then harvested. Cell lysates were prepared, resolved by SDS-12% PAGE, and probed with antibodies against Tax, cyclin B1, Skp2, p27, p21, I-κBα, and β-actin (Actin), respectively.

To investigate the mechanism by which Tax induces senescence, we analyzed five well-characterized Tax mutants. Tax^V89A^ and Tax^L319R, L320S^ (M47) are deficient in HTLV-1 LTR *trans*-activation, but competent in NF-κB activation [22], [23]. By contrast, Tax^H43Q^ and Tax^T130A, L131S^ (M22) are defective in NF-κB activation, but are able to *trans*-activate the HTLV-1 LTR [23], [24]. Finally, Tax^K85A^ mutant is inactive for both [22]. We use the degradation of I-κBα as an indicator of NF-κB activation and the decrease in cyclin B1 and Skp2 levels with concomitant increase in the levels of p21 and p27 as surrogate markers for senescence induction. HeLa cells were transduced with lentivirus vectors carrying each of the Tax alleles or the control GFP gene, and analyzed by immunoblots. As shown in Fig. 1, wild-type Tax (WT) and those Tax mutants (V89A and M47) that are potent activators of NF-κB greatly reduced the levels of cyclin B and Skp2, and dramatically elevated p21 and p27 expression. Tax mutants that are impaired in NF-κB activation (H43Q, K85A, and M22), in comparison, had only moderate or no effect on cyclin B, Skp2, p21, or p27. These data suggest a link between Tax-IRS and persistent NF-κB activation.

### NF-κB inhibition by a degradation-resistant I-κBα mutant prevents Tax-induced rapid senescence

**Figure 2:**
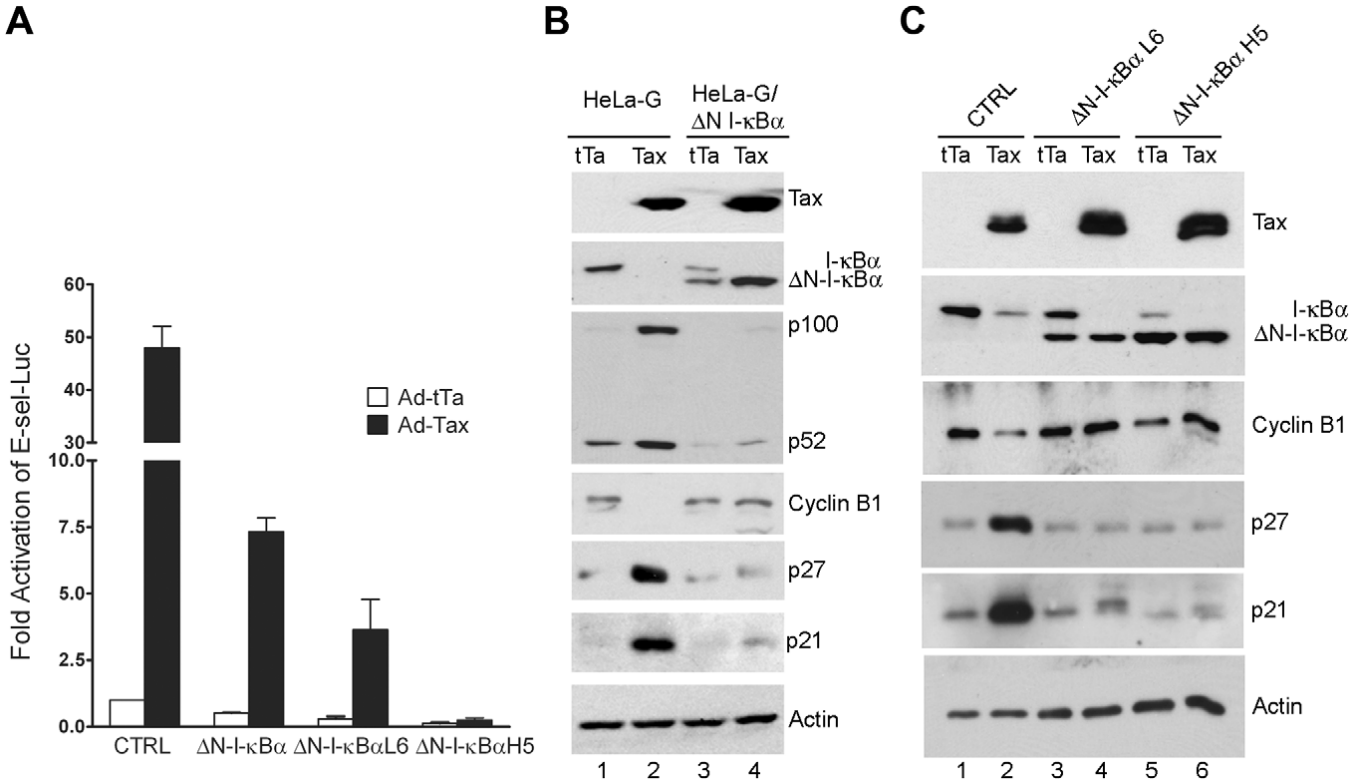
ΔN-I-κBα, a degradation-resistant form of I-κBα, blocks Tax-mediated activation of NF-κB, induction of p21 and p27, and down-regulation of cyclin B. HeLa-G cells stably expressing ΔN-I-κBα were derived after lentivirus-mediated transfer of the ΔN-I-κBα gene (see Materials and Methods) as a pool (ΔN-I-κBα) or as isolated clones that express ΔN-I-κBα at a level lower (ΔN-I-κBα L6) or higher (ΔN-I-κBα H5) than that of endogenous I-κBα. (**A**) E-selectin-Luc, an NF-κB luciferase reporter plasmid, and the control *Renilla* luciferase reporter plasmid, pRL-TK, were transfected into the cell lines listed above. One day post-transfection, the medium was changed and the transfected cells were infected by Ad-Tax or Ad–tTa (a control adenovirus vector for the *tet* trans-activator) at an MOI of 10. Cells were harvested 24 hours after infection. The firefly luciferase activity was measured and normalized against *Renilla* luciferase activity. Data (mean ± standard deviation) are from at least three independent experiments. Fold activation of the promoter by Tax in each cell line was determined by comparing the normalized luciferase activities to that of the Ad-tTa-transduced control HeLa-G cells. (**B & C**) Asynchronously grown HeLa-G and pooled HeLa-G/ΔN-I-κBα cells (**B**) or HeLa-G and HeLa-G/ΔN-I-κBα cell lines L6 and H5 (**C**) were transduced by Ad-Tax or At-tTa at an MOI of 10. Cell lysates were prepared at 48 (**B**) or 40 hours (**C**) post-transduction for immunoblots using the antibodies indicated.

We next asked if direct inhibition of NF-κB could prevent Tax-IRS. To this end, we used a lentivirus vector to derive HeLa cell lines that expressed ΔN-I-κBα, a truncated form of I-κBα lacking 36 NH_2_-terminal amino acid residues that constitute the IKK phosphorylation and degradation domain of I-κBα [25]. This deletion renders ΔN-I-κBα resistant to IKK-mediated degradation. As a result, ΔN-I-κBα constitutively retains NF-κB in the cytoplasm and blocks NF-κB activation [26]. A pool of ΔN-I-κBα-expressing HeLa-G cells (HeLa-G/ΔN-I-κBα) and two cloned cell lines: HeLa-G/ΔN-I-κBα L6 and H5 were generated. HeLa-G/ΔN-I-κBα L6 and H5 respectively express ΔN-I-κBα at lower (L6) and higher (H5) levels than that of the endogenous I-κBα. As expected, E-selectin-Luc reporter assays indicated that NF-κB activation by Ad-Tax (an adenovirus vector for Tax) versus Ad-tTa (an adenovirus vector for the *tet* trans-activator) control in the HeLa-G/ΔN-I-κBα cell lines were repressed, and the degrees of repression correlated with the extent of ΔN-I-κBα expression (Fig. 2A). We noticed that HeLa-G/ΔN-I-κBα cells in general grew more slowly and took longer to attach to a culture dish after plating. These phenotypes reflect the role of basal NF-κB activity in growth, survival, and expression of adhesion molecules. Efforts to establish ΔN-I-κBα-expressing human T cell lines were unsuccessful however, most likely because of the importance of NF-κB for T cell proliferation and survival.

HeLa-G (CTRL), HeLa-G/ΔN-I-κBα, HeLa-G/ΔN-I-κBα L6, and HeLa-G/ΔN-I-κBα H5 cells were then transduced with Ad-Tax and Ad-tTa vectors respectively, and analyzed for their levels of Tax, I-κBα cyclin B, p21, p27, and actin. As shown in Fig. 2B & C, 48 hours after transduction by Ad-Tax, the endogenous wild-type I-κBα level became greatly reduced in both parental HeLa-G (CTRL) and HeLa/ΔN-I-κBα cells due to Tax-mediated IKK activation (Fig. 2B lanes 2 & 4, Fig. 2C lanes 2, 4 & 6). As expected, Tax was unable to induce ΔN-I-κBα degradation (Fig. 2B lane 4, Fig. 2C lanes 4 & 6). Rather, the level of ΔN-I-κBα was increased by Tax. This is because the *cis* regulatory element used for ΔN-I-κBα expression is a composite of the HIV LTR and the CMV immediate early promoter known to be activated by Tax. ΔN-I-κBα also blocked the induction and processing of the NF-κB2 precursor, p100, by Tax (compare Fig. 2B lanes 2 & 4), thereby dampening activation of the alternative NF-κB pathway. Importantly, while Tax greatly elevated the levels of p21 and p27 in the parental HeLa-G cells (compare Fig. 2B lanes 1 & 2, and Fig. 2C lanes 1 & 2), little change in p21 or p27 was observed in HeLa-G/ΔN-I-κBα cells (compare Fig. 2B lanes 3 & 4, and Fig. 2C lanes 3 & 4 and 5 & 6). At 48 hours after Ad-Tax transduction, most if not all cell cycle activities ceased for HeLa-G cells as indicated by the complete absence of cyclin B1 (Fig. 2B lane 2). By contrast, HeLa/ΔN-I-κBα cells continued to produce normal levels of cyclin B1 irrespective of Tax (Fig. 2B lane 4, Fig. 2C lanes 4 & 6), suggesting that their cell cycle progression is not significantly influenced by Tax.

**Figure 3:**
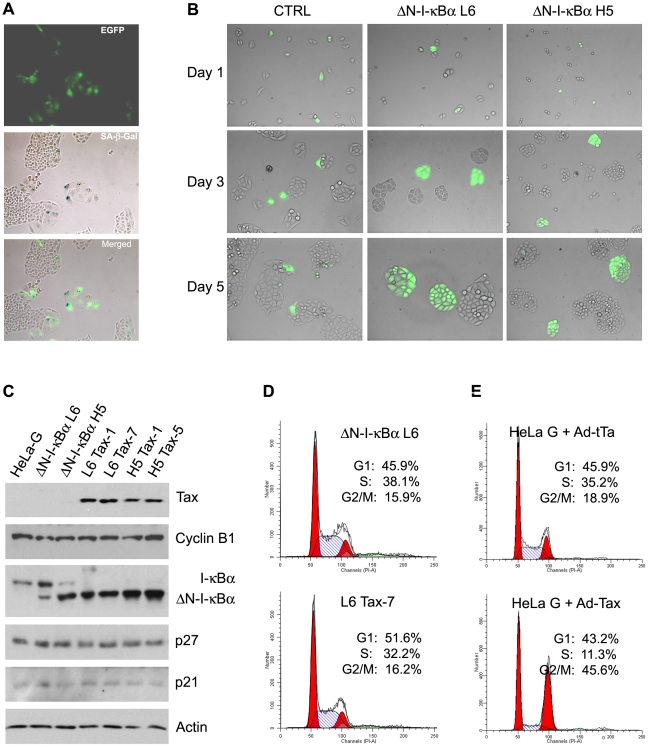
ΔN-I-κBα prevents Tax-induced senescence. (**A**) HeLa-G cells become senescent 5 days post-transduction by Ad-Tax. Twenty five thousand HeLa-G cells were plated on a 3.5-cm dish, transduced with Ad-Tax at an MOI of 1 for 5 days, and then fixed and stained with X-gal under acidic condition [14], [18]. Cells were photographed for EGFP expression (EGFP) using fluorescence microscopy and with an RGB filter under visible light for senescence β-galactosidase expression (SA-β-Gal). The EGFP and RGB images were overlaid (merged) to show correlation of EGFP and SA-β-Gal expression. (**B**) ΔN-I-κBα prevents Tax-induced senescence. Asynchronously grown HeLa-G (labeled as CTRL) or HeLa-G/ΔN-I-κBα cells (ΔN-I-κBα L6 and ΔN-I-κBα H5) were transduced by Ad-Tax at an MOI of 1 such that approximately 30–50% of cells were transduced. They were sparsely plated as single cells. Cells were photographed at 1, 3, and 5 days after Ad-Tax transduction as indicated. EGFP-positive cells were Ad-Tax-transduced. EGFP-negative cells were un-transduced and served as an internal control. (**C**) Establishment of stable Tax-expressing HeLa/ΔN-I-κBα cell lines. Two HeLa-G/ΔN-I-κBα/Tax cell lines each (L6 Tax-1 and Tax-7; and H5 Tax-1 and Tax-5) were derived from their parental cell lines ΔN-I-κBα L6 and ΔN-I-κBα H5 after transduction with a Tax lentivirus vector, LV-Tax [14]. Cell lysates from HeLa-G, ΔN-I-κBα L6, ΔN-I-κBα H5, L6 Tax-1, L6 Tax-7, H5 Tax-1 and H5 Tax-5 were resolved by SDS-12% PAGE and immunoblotted with the antibodies indicated on the right. (**D**) Flow cytometry histograms of propidium iodide-stained HeLa-G/ΔN-I-κBα L6 (upper panel) and its Tax-expressing counterpart L6 Tax-7 cell lines (lower panel). The percentages of cell population in G1, S, and G2/M phases of the cell cycle were determined as described in Materials and Methods. (**E**) Flow cytometry histograms of Ad-Tax- and Ad-tTa-trasnsduced HeLa-G cells. HeLa-G cells were plated at a density of 2×10^5^ cells/well in a 6-well plate, transduced with Ad-Tax or Ad-tTa at an MOI of 10 for 48 hours. Cell preparation and data analyses are as described in (**D**).

HeLa-G/ΔN-I-κBα cells and their parental cell line, HeLa-G (abbreviated from HeLa/18×21-EGFP), all contain a reporter cassette, 18×21-EGFP, composed of the enhanced green fluorescent protein (EGFP) gene transcriptionally regulated by 18 copies of the 21-bp-repeat Tax-responsive enhancer element [27]. Cells harboring the reporter cassette synthesize little EGFP when untreated, but produce it abundantly when infected by an adenovirus (Ad-Tax) or a lentiviral vector (LV-Tax) for Tax, thereby rendering Tax-expressing cells to be easily scored microscopically [18], [27]. To demonstrate further that Tax-IRS can be prevented by blocking NF-κB activation, HeLa-G, HeLa-G/ΔN-I-κBα L6 and HeLa-G/ΔN-I-κBα H5 cells were plated sparsely on a culture dish. Cells were then transduced with Ad-Tax at a multiplicity of infection (MOI) of approximately 1 (viral titer determined on HEK 293 cells) such that 30–50% of the population became transduced, and then monitored for 5 days. As shown in Fig. 3A, Tax-expressing EGFP-positive HeLa-G cells became enlarged, flattened, highly vacuolated, and expressed the senescence-associated β-galactosidase over 5 days. The nuclei of the senescent cells also became significantly larger as previously reported [14], [18]. Soon after Ad-Tax transduction, EGFP-positive (Tax-expressing) HeLa-G cells ceased proliferation (Fig. 3B left column). By contrast, neighboring EGFP-negative cells divided 5–6 times over 5 days to form sizable colonies of approximately 40–50 cells. Importantly, Tax-expressing HeLa-G/ΔN-I-κBα L6 and H5 cells continued to grow and divide (Fig. 3B middle and right columns), albeit their rate of cell division was slightly reduced compared to the un-transduced cells, with most colonies smaller in size and containing 14–30 cells (see Fig. 3 Day 5 middle and right panels). After 5 days in culture, the EGFP expression in the dividing Ad-Tax-transduced colonies decreased moderately and sometimes became variegated. This is likely a result of the transient nature of Ad-vector-driven Tax expression and the dilution of Tax upon successive cell divisions. Interestingly, as long as ΔN-I-κBα was expressed at a level comparable to that of the endogenous I-κBα (Fig. 3B, ΔN-I-κBα L6), Tax-IRS could be effectively prevented (Fig. 3B and supplemental Fig. S3A). No advantage was seen with a higher level of ΔN-I-κBα expression (Fig. 3B, ΔN-I-κBα H5). This suggests that once a threshold level of ΔN-I-κBα sufficient to block NF-κB is reached, additional ΔN-I-κBα has little biological impact. Finally, HeLa-G/ΔN-I-κBα L6 and H5 cells were transduced with a lentivirus vector for Tax. Indeed, stable Tax expression was readily established in these cells (Fig. 3C). In HeLa-G/ΔN-I-κBα/Tax cell lines (L6 Tax-1 L6 Tax-7, H5 Tax-1, and H5 Tax-5), endogenous I-κBα was undetectable, consistent with its degradation after IKK activation by Tax. Their levels of cyclin B1, p27, and p21, however, were similar to the Tax-null parental cell lines (Fig. 3C, HeLa-G, ΔN-I-κBα L6, and ΔN-I-κBα H5). Finally, the flow cytometry DNA histogram of the L6 Tax-7 cell line was similar to that of the parental ΔN-I-κBα L6 cell line except for a small increase and a corresponding decrease of cells in the G1 and the S phases respectively (Fig. 3D), reflecting slower growth of Tax-expressing cells, but in stark contrast to the dramatic change seen in Tax-expressing HeLa-G cells (Fig. 3E and previous publications [14], [18]). It should be pointed out that time-lapse microscopy of reporter cells expressing fluorescent-ubiquitin-cell-cycle indicators [28] has revealed that many Ad-Tax-transduced cells completed S/G2, but bypassed mitosis and became arrested in G1/senescence, but with G2/M DNA content (Yang et al. manuscript in revision). These results demonstrate that Tax-induced senescence is NF-κB-driven and suggest that many of the cell cycle abnormalities induced by Tax may also be caused by NF-κB. Once NF-κB is blocked by ΔN-I-κBα, constitutively activated IKK has minimal impact on cell cycle progression or senescence.

### Hyper-activated NF-κB stabilizes p27 protein and p21 mRNA

**Figure 4:**
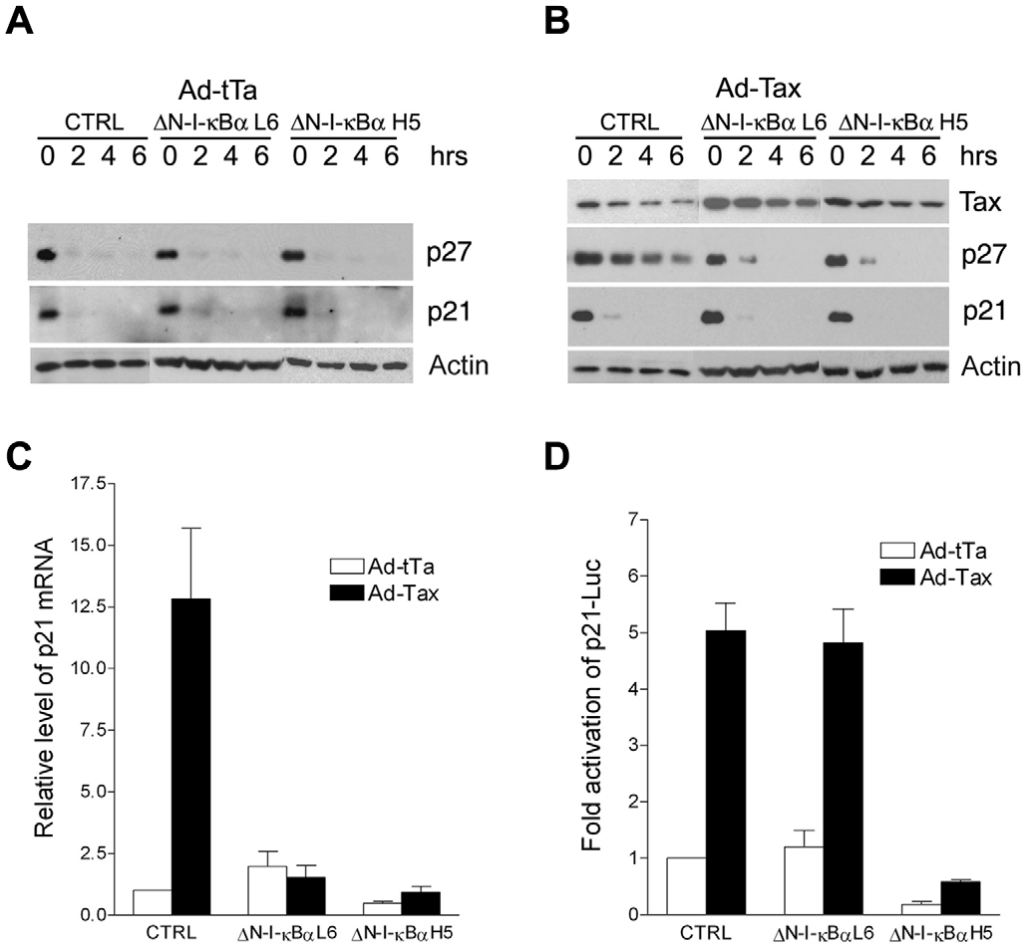
Inhibition of NF-κB prevents the stabilization of p27 protein and the up-regulation of p21 mRNA, but not the trans-activation of p21 promoter by Tax. (**A**) P21 and p27 protein levels in Ad-tTa-transduced HeLa-G (CTRL), ΔN-I-κBα L6, and ΔN-I-κBα H5 cells. Times (0, 2, 4 and 6 hours) after the addition of protein synthesis inhibitor, cycloheximide (100 µg/ml), are indicated. Immunoblots for p21 and p27 were done with all samples loaded in one gel. The comparable loading of protein samples in each set of 4 time points was confirmed by an actin immunoblot, which was sometimes done separately before the p21 and p27 blots were performed. (**B**) P21 and p27 protein levels in Ad-Tax-transduced parental HeLa-G (CTRL), ΔN-I-κBα L6, and ΔN-I-κBα H5 cells. Immunoblots were carried out as in (A) except the amount of lysate used in each lane of the control (CTRL) was one fifth that of the ΔN-I-κBα cell lines because of the dramatic p21 and p27 up-regulation by Tax in the former. (**C**) Quantitative analysis of p21 mRNA in Ad-tTa- (open bars) versus Ad-Tax- (solid bars) transduced HeLa-G (CTRL), ΔN-I-κBα L6, and ΔN-I-κBα H5 cells. (**D**) Transcriptional activation of p21-Luc reporter (−2.4 kB to +16 of p21 promoter fused to Luc) by Ad-tTa versus Ad-Tax in HeLa-G (CTRL), ΔN-I-κBα L6, and ΔN-I-κBα H5 cells. DNA transfection, luciferase assays and data analyses are as in Fig. 3A.

We have shown recently that Tax stablizes p27 protein in part by causing APC/C-mediated poly-ubiquitination and degradation of Skp2, the substrate-recognition subunit of SCF^Skp2^, the E3 ubiquitin ligase that targets p27 for destruction [14]. Tax also up-regulates p21 expression by promoter *trans*-activation and mRNA stabilization [17]. Since NF-κB inhibition prevented the up-regulation of p21 and p27 by Tax, we asked if p27 protein stabilization and p21 mRNA stabilization were also NF-κB-driven. The stabilities of p21 and p27 proteins in HeLa-G and its ΔN-I-κBα progenies L6 and H5 were similar after infection with the control Ad-tTa vector, (Fig. 4A) suggesting that NF-κB in HeLa-G cells was already regulated by endogenous I-κBα, additional blocking of NF-κB with ΔN-I-κBα did not alter the levels of p21 and p27. By contrast, the stabilization of p27 protein and the increase of p21 mRNA (through stabilization) caused by Tax were abrogated by ΔN-I-κBα (Fig. 4B & C, compare HeLa-G control, CTRL, with its ΔN-I-κBα-expressing progenies L6 and H5). *Trans*-activation of the p21 promoter by Tax was unaffected by NF-κB inhibition except for a reduction in overall luciferase reporter activities in ΔN-I-κBα H5 cells (Fig. 4D). This is as might be expected if NF-κB mediates the stabilization of p21 mRNA. These data support the notion that NF-κB is the upstream effector of the increase in p21 mRNA stability and APC/C-mediated p27 protein stabilization.

### RelA mediates senescence induction by Tax

**Figure 5:**
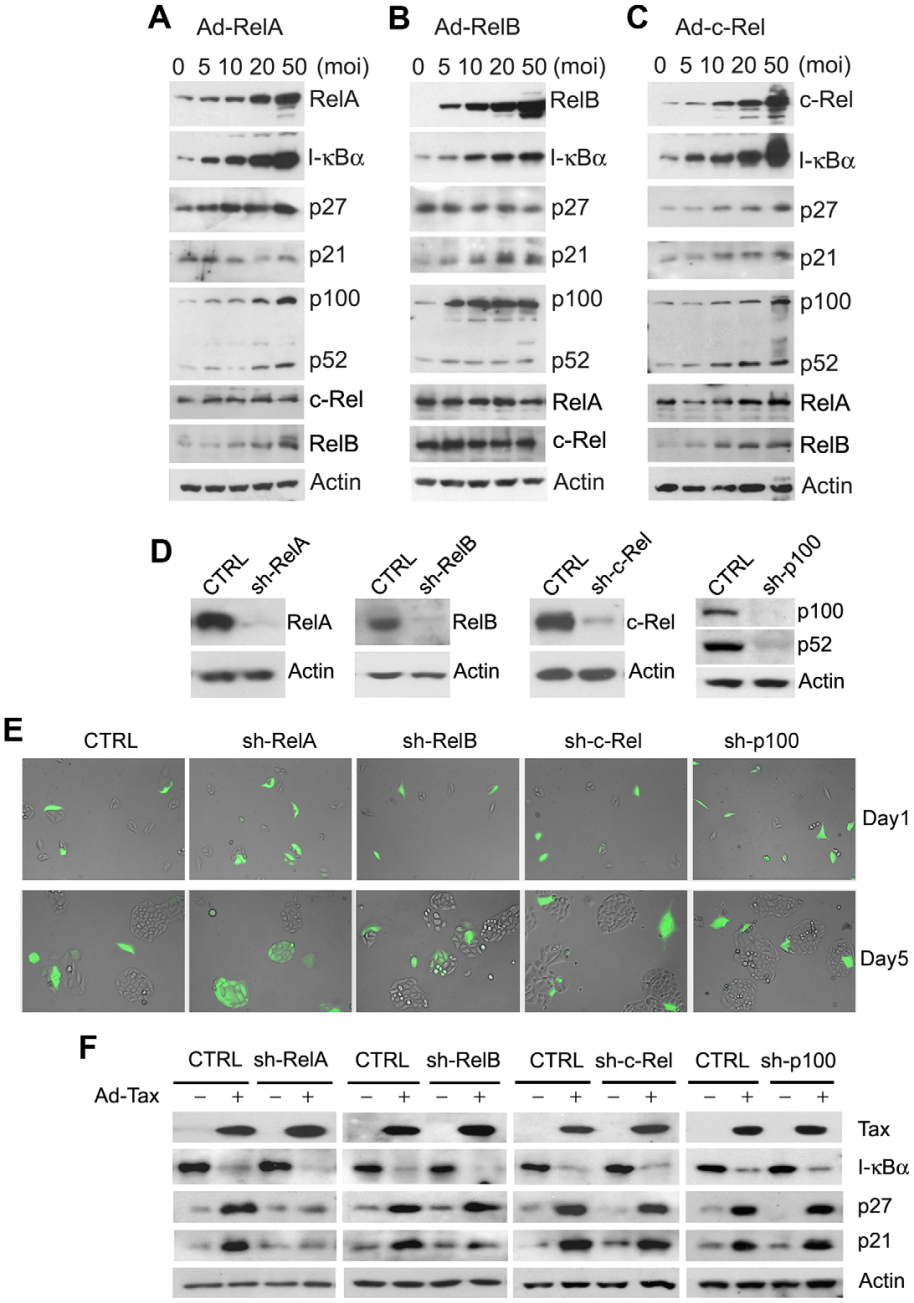
RelA is the major downstream effector of Tax-induced senescence. (**A–C**) The effects of RelA, RelB, and C-Rel over-expression. HeLa-G cells were transduced with increasing amounts (MOI = 0, 5, 10, 20 and 50) of Ad-RelA, Ad-RelB, or Ad-c-Rel vectors for 48 hours. Cell lysates were prepared and analyzed by immunoblotting using the antibodies indicated. (**D**) Immunoblot analysis of parental HeLa-G cell line (CTRL) and progeny cell lines (sh-RelA, sh-RelB, sh-c-Rel, and sh-p100) wherein RelA, RelB, c-Rel and p100 had been knocked down respectively by small hairpin RNAs (shRNAs) stably expressed after lentivirus vector transduction and puromycin selection. The sequences targeted by the shRNAs are as listed (supplemental Table S3) (**E**) RelA knockdown prevented Tax-induced senescence. Cell lines from (D) were transduced with Ad-Tax and photographed as in Fig. 3B at days 1 and 5. (**F**) Immunoblot analysis of lysates from cell lines in (D) prepared 48 hours after transduction by Ad-Tax at an MOI of 10. CTRL denotes parental HeLa-G cell line. Progeny cell lines with various NF-κB/Rel genes knocked down are as indicated.

As Tax activates all NF-κB/Rel family members including RelA, c-Rel, RelB, p50 and p52, we ask which NF-κB/Rel member(s) is/are responsible for Tax-IRS. To this end, we over-expressed RelA, RelB, and c-Rel, the three NF-κB/Rel members that contain *trans*-activation domains by transducing HeLa-G cells with adenovirus expression vectors: Ad-RelA, Ad-RelB, and Ad-c-Rel respectively. RelA dramatically activated the E-selectin-Luc NF-κB reporter construct in a dose-dependent manner as expected (supplemental Fig. S1A). Since I-κBα mRNA transcription is under NF-κB control, the levels of I-κBα in Ad-RelA-transduced cells also increased sharply in accordance with RelA levels (Fig. 5A). Likewise, the levels of RelB, p100, and p52 were also elevated in a dose-dependent manner in response to RelA (Fig. 5A), indicating that RelA is a critical activator of the alternative NF-κB pathway. Surprisingly, except for a small increase in p27, no dramatic change in p21 or cyclin B level was detected in cells over-expressing RelA (Fig. 5A), and no overt cellular senescence was observed (supplemental Fig. S1B). RelB and to a lesser extent, c-Rel, increased p21 expression. C-Rel, but not RelB, further raised p27 level (Fig. 5B & C). These increases in p21 and p27 levels are relatively modest when compared to those caused by Tax however. Similar to RelA, c-Rel dramatically induced I-κBα expression. By contrast, RelB had only a moderate effect on I-κBα, but significantly induced the expression, but not the processing of p100, which is expected to bind and retain RelB in the cytoplasm in its inactive form (Fig. 5B). Finally, c-Rel also increased p100 expression (Fig. 5A & C) and elevated RelB levels, but had little effect on RelA and vice versa (Fig. 5A). No overt senescence was observed for cells over-expressing RelB and c-Rel (supplemental Fig. S1B).

While these data suggest that the classical NF-κB pathway, when activated, can positively affect p100 expression and processing as reported recently [29] and that RelA and c-Rel can up-regulate p27 while RelB, p21, the effects of RelA, RelB and c-Rel on p21 and p27 induction were modest most likely because their activities were quickly dampened by the surge in I-κBα and p100 (Fig. 5A–C). As such, these results were inconclusive in implicating a specific NF-κB/Rel family member as the key mediator of Tax-induced senescence. For this reason, stable expression of small hairpin RNA (shRNA) in HeLa-G cells was carried out to knockdown RelA, RelB, c-Rel, and p100 respectively (Fig. 5D). Most interestingly, knockdown of RelA and to a rather limited extent, that of RelB, but not that of c-Rel or p100 prevented Tax-induced senescence (Fig. 5E and supplemental Fig. S3B). This is evidenced by the proliferation of EGFP+ HeLa-G/RelA^KD^ cells (Fig. 5E sh-RelA Day 5) and the absence of p21 and p27 up-regulation therein after Ad-Tax transduction (Fig. 5F, sh-RelA). We observed limited cell division of EGFP+ HeLa-G/RelB^KD^ cells after Tax expression (sh-RelB panels of Fig. 5E). This partial effect correlated with the muted p21 but highly robust p27 induction by Tax in HeLa-G/RelB^KD^ cells (sh-RelB panels 5F). These results demonstrate RelA to be the major effector of Tax-IRS. Furthermore, the facts that RelB knockdown prevents Tax/RelA-/NF-κB-induced p21 increase and RelA can up-regulate RelB expression (Fig. 5A) indicate that RelB acts downstream of RelA to stabilize p21 mRNA (Fig. 5F).

### HBZ mitigates senescence induction by Tax

The 3′ region of HTLV-1 encodes an mRNA transcript of opposite polarity to the major HTLV-1 mRNA. This anti-sense mRNA is singly spliced and encodes a basic domain-leucine zipper protein known as the HTLV-1 b-Zip (HBZ) protein [20]. Earlier studies have indicated that HBZ could down-regulate Tax-mediated *trans*-activation of the HTLV-1 LTR by binding CREB, c-Jun, and CBP/p300 [20], [21]. Most recently, HBZ has been shown to selectively inhibit the classical NF-κB pathway by blocking RelA binding to DNA and by targeting RelA for ubiquitin-mediated degradation [19]. That HBZ antagonizes the activities of Tax by dampening LTR *trans*-activation and NF-κB activation, and the results herein showing that NF-κB hyper-activation by Tax triggers senescence raise the possibility that HBZ may prevent Tax-IRS. Indeed, in agreement with published results, NF-κB and LTR activation by Tax are both attenuated by HBZ (supplemental Fig. S2A & B). The HBZ TTG mutant wherein the HBZ translational initiation site ATG had been mutated to TTG failed to block NF-κB or LTR activation by Tax (supplemental Fig. S2A & B).

**Figure 6:**
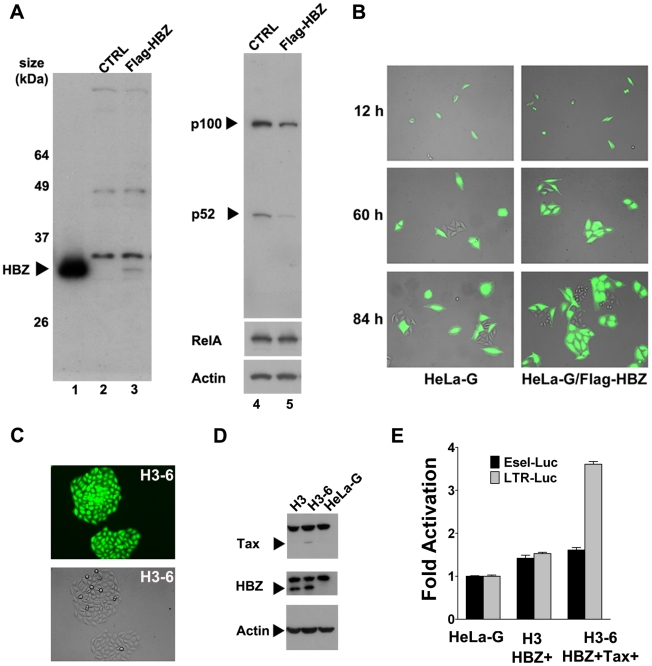
HTLV-1 HBZ mitigates cellular senescence induced by Tax. (**A**) Immunoblot analysis of HeLa-G/Flag-HBZ cells. HeLa-G cells transduced by the pBabe-Flag-HBZ retroviral vector were selected in liquid media containing 1 µg/ml puromycin for 6 days. Puromycin-resistant cells were further subcloned by limiting dilution. HEK293T cells transfected with the pBabe-Flag-HBZ plasmid, control HeLa-G cells (CTRL), and HeLa-G/Flag-HBZ cells were harvested for immunoblots using the Flag epitope antibody (lanes1, 2 & 3 respectivley). HeLa-G (CTRL) and HeLa-G/Flag-HBZ samples were further probed with antibodies against p100/p52/NF-κB2, RelA, and β-actin (lanes 4 & 5). (**B**) Tax-induced rapid senescence is delayed in HeLa-G/Flag-HBZ cells. A representative HeLa-G/Flag-HBZ cell line and its parental HeLa-G cell line were transduced by Ad-Tax as in Fig. 3B, and photographed at 12, 60, and 84 hours post-transduction. (**C**) HeLa-G/Flag-HBZ cells can stably express Tax. HeLa-G/Flag-HBZ cells were transduced with the Tax lentivirus vector, LV-Tax, as in Fig. 3C, and then sorted for high EGFP expression using a BD FACSAria cell sorter, which deposited one cell per well in 96-well plates. Cell clones were grown and expanded in the presence of 200 µl of DMEM containing 20% FBS, 2 mM L-glutamine, and antibiotics. The fluorescence and phase contrast images (upper and middle panels) of a representative clone, H3-6, are shown. (**D**) Immunoblots of HeLa-G, H3 and H3-6 cell lines. Lysates of HeLa-G/Flag-HBZ (H3), HeLa-G/Flag-HBZ/Tax (H3-6), and control HeLa-G cells were immunoblotted with antibodies against Tax, Flag-HBZ, and β-actin (Actin), respectively. (**E**) NF-κB and LTR reporter activities in HeLa-G, H3, and H3-6 cells. E-selectin-Luc and LTR-Luc respectively were co-transfected with the internal control *Renilla* luciferase plasmid, pRL-TK, into the indicated cell lines in triplicates. Luciferase activities were measured, normalized, and fold *trans*-activation calculated as in Fig. 2A.

To determine if HBZ can mitigate or prevent Tax-IRS, a Flag-epitope-tagged HBZ cDNA construct was generated by PCR using the cDNA of the spliced HBZ mRNA as template, and inserted into the pBabe-Puro murine retroviral vector [30] to yield pBabe-Flag-HBZ. Like native HBZ, Flag-HBZ efficiently inhibits NF-κB activation by Tax (supplemental Fig. S2C). HeLa-G cells expressing Flag-HBZ (HeLa-G/Flag-HBZ) were then derived after retroviral gene transfer and puromycin selection, and confirmed for HBZ expression by immunoblotting (compare Fig. 6A lanes 2 & 3). Consistent with the notion that HBZ inhibits NF-κB, the level of p100, an endogenous NF-κB-inducible gene (see Fig. 5A), was lowered in HBZ-expressing cells (compare Fig. 6A lanes 4 & 5). This is in contrast to the previous report that HBZ down-regulates only the classical but not the alternative NF-κB pathway [19]. No significant change in the level of RelA was detected in HBZ-expressing cells (Fig. 6A). Thus, the inhibition of NF-κB by HBZ is likely due more to a direct inhibition of NF-κB binding to DNA and less to HBZ-induced RelA degradation. Importantly, a significant number of HeLa-G/Flag-HBZ cell clones transduced by Ad-Tax continued to divide up to 3–4 cell division cycles but eventually ceased proliferation, indicating that Ad-Tax-induced senescence was delayed by HBZ (Fig. 6B). The degree of senescence resistance of HeLa-G/Flag-HBZ is approximately 25% (supplemental Fig. S3C), hence no dramatic change in p21 and p27 up-regulation was observed (not shown). Finally, stable cell lines (H3-6 HBZ+Tax+ is shown as an example) that express low levels of Tax could be established in HeLa-G/Flag-HBZ cells (H3 HBZ+) after transduction by the Tax lentivirus vector, LV-Tax (Fig. 6C & D). While NF-κB activation in these cells was undetectable due to HBZ and a low level of Tax expression (Fig. 6E, E-selectin-Luc reporter assay), the 18×21-EGFP reporter remained significantly induced (see Fig. 6C fluorescence image) and LTR-Luc reporter trans-activation readily detected (Fig. 6E, compare H3 HBZ+ and H3-6 HBZ+Tax+). These results support the notion that HBZ can moderate NF-κB activation by Tax and thereby delay or prevent the onset of senescence. This in turn allows cells expressing low to moderate levels of Tax to grow, divide, and produce viral particles. Importantly, these results strongly suggest that in order for NF-κB hyper-activation by Tax to drive pre-leukemic or leukemic cell growth, inactivation of the senescence checkpoint is still necessary.

## Discussion

The data reported herein have revealed constitutive IKK activation and NF-κB hyper-activation to be the cause for Tax-induced cellular senescence. These results have further established RelA to be the major driver of cellular senescence. Because NF-κB activation is often associated with cell survival and proliferation, we previously thought that a mechanism distinct from IKK/NF-κB activation is responsible for the cellular senescence induced by Tax [31]. Present data show unequivocally that the potentially deleterious IKK/NF-κB hyper-activation by Tax (or other means) immediately triggers a checkpoint mediated by p21 and p27, which act independently of pRb and p53 to induce cellular senescence [14].

We have previously reported that the stabilization of p27 by Tax is a result of premature activation of the APC/C, which brings about degradation of Skp2 and inactivation of SCF^Skp2^, the E3 ligase that targets p27 for degradation [14]. Present data indicate that hyper-activated NF-κB acts upstream of APC/C to stabilize p27. How NF-κB (RelA specifically) affects unscheduled activation of the APC/C remains to be elucidated. In agreement with our results, the loss of Skp2 has been shown recently to facilitate p21-/p27-mediated and Arf/p53-independent senescence in Skp2-/p19Arf-null mice [32].

The dramatic induction of p21 mRNA by Tax is a result of both promoter *trans*-activation and mRNA stabilization [17]. The increase in p21 mRNA stability is now shown to be RelB-dependent. Since RelB expression is up-regulated by RelA [29] (also see Fig. 5A), repression or knockdown of RelA effectively prevents p21 mRNA increase. How RelB increases p21 mRNA stability is not clear at present. It may promote the expression of a cellular factor crucial for blocking p21 mRNA degradation.

We think p21-/p27-mediated senescence constitutes a checkpoint for blocking potentially oncogenic IKK-NF-κB hyper-activation. Since NF-κB is constitutively active in many human cancers, especially leukemia, present data would suggest that cellular changes that disable this senescence pathway must accompany NF-κB de-regulation in human cancers. Indeed, impairments of p21 and p27 function and expression have been observed in most if not all HTLV-1-transformed T cells previously [14], [33]. Studies of human breast cancers also show a correlation between the over-expression of IKK (i.e. chronic NF-κB activation) and the cytoplasmic localization of p21, in support this conclusion [34]. We think Tax-induced cell cycle aberrations such as DNA damage, and chromosome instability is likely to be NF-κB-mediated. That HeLa-G/ΔN-I-κBα/Tax cell lines are largely normal in their growth and division is consistent with this conclusion.

Cellular signaling pathways activated by oxidative stress, inflammation, and DNA damage are known to cause aging and shortened lifespan, and these pathways impact NF-κB activation [35]. Earlier studies have also implicated NF-κB/Rel in aging, cell cycle arrest, or senescence [36]–[39]. Senescent cells are known to secret chemo-attractants for macrophages and NK cells [40], which are responsible for their eventual elimination. Whether senescent HTLV-1-infected cells may use this mechanism for virus transmission and spread, especially to macrophages, can only be speculated upon at this point.

Our results also show that NF-κB activation caused by the over-expression of RelA, RelB, or c-Rel, however potent it may be, occurs transiently and leads rapidly to a steep surge in I-κBα and p100 (and possibly other I-κB proteins), which promptly dampen NF-κB activity and prevent drastic p21/p27 up-regulation and senescence. This contrasts with the NF-κB hyper-activation by Tax that is mediated by the destruction of I-κBα and I-κB-like molecules via activated IKKs.

**Figure 7:**
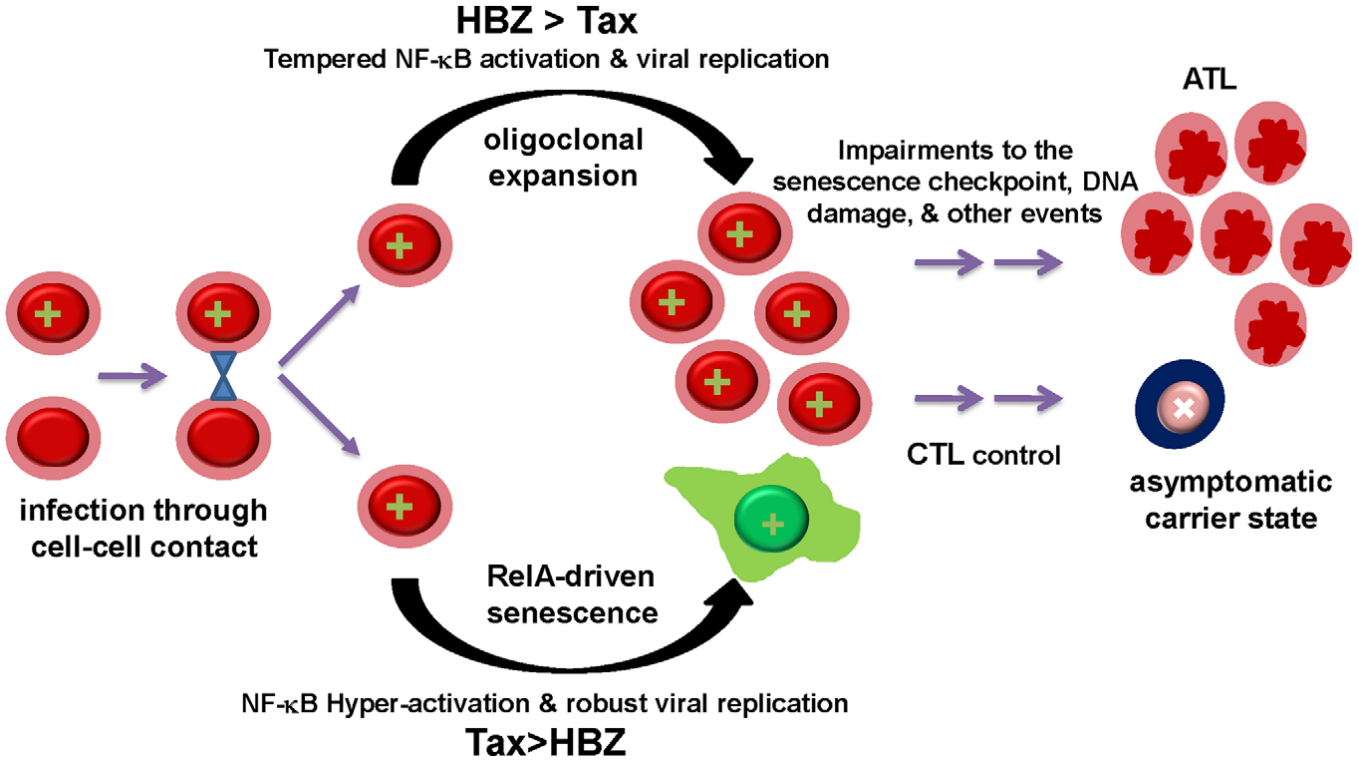
The balance between Tax and HBZ expression regulates the outcome of HTLV-1 infection. HTLV-1 infection may lead to two alternative outcomes. Proviral integration sites and transcription factors availability determine the levels of Tax and HBZ expression in infected cells. High levels of Tax drive NF-κB hyper-activation and robust viral replication. Dysregulated RelA then induces APC/C- and RelB-mediated up-regulation of p27 and p21, and cellular senescence. By contrast, HBZ modulates viral *trans*-activation and NF-κB activation by Tax. Moderated NF-κB activation and other mitogenic activities of Tax, in turn, promote survival and oligoclonal expansion of HTLV-1-infected T cells. Cytotoxic T lymphocyte killing can control virus replication in asymptomatic carriers and select for cells that carry latent proviral DNA. Finally, genetic or epigenetic changes that impair the p21-/p27-senescence checkpoint may allow persistent Tax expression, NF-κB hyper-activation, and facilitate development of Tax-independent NF-κB activation, leading to ATL.

How does HTLV-1 infection lead to adult T-cell leukemia when the expression of Tax causes senescence? Based on our results, moderation of Tax activities by HBZ, especially at the level of NF-κB activation, is obligatory for the proliferation and expansion of infected cells. Thus, the balance between Tax and HBZ expression, determined by the sites of proviral integration and the availability of cellular transcription factors, can modulate the extent of viral replication and NF-κB activation during infection. This, in turn, may determine the fate of infected cells, which likely ranges from robust viral replication and senescence, oligoclonal expansion, to viral latency and persistence (Fig. 7). Recently, Tax has been shown to up-regulate the expression of HBZ and this up-regulation is influenced by the HTLV-1 integration sites [41]. Furthermore, HBZ has been found to enhance viral infectivity and persistence, and facilitate proliferation of HTLV-1-infected lymphocytes [42], [43]. Finally, HBZ mRNA expression has been shown to correlate with HTLV-1 proviral load while Tax expression was found to decline over time after HTLV-1-infection in a rabbit model [44]. These results are consistent with the notion that HBZ plays a critical role in viral persistence in part by down-regulating the senescence-inducing activity of Tax (Fig. 7). Finally, Tax-induced senescence should be considered as a precancerous condition. While HBZ may either delay or prevent the onset of senescence by attenuating the expression of Tax and NF-κB activation by Tax, cellular changes that inactivate the senescence checkpoint may ultimately facilitate potent and persistent NF-κB activation by Tax to promote leukemia development.

ATL cells often cease to express Tax, but continue to produce HBZ mRNA and, presumably, HBZ protein [45]. Whereas Tax, in association with HBZ, may facilitate cell proliferation and survival in the early stage of HTLV-1 infection, at a later time, cytotoxic T lymphocyte killing of Tax-expressing cells likely select for loss of Tax expression. It is conceivable that defects in the senescence checkpoint that develop during the evolution of the infected cells then facilitate the emergence of Tax-independent NF-κB activation (Fig. 7). Finally, the mitogenic activity of HBZ mRNA [46] may also play a role in leukemia maintenance.

## Materials and Methods

### Immunoblotting

Standard methods were used for immunoblotting and flow cytometry. Each sample typically contains 20-30 µg of whole cell proteins. HTLV-1 Tax hybridoma antibody 4C5 was as described [14]. The rabbit polyclonal antibody against HBZ was a kind gift of Dr. Jean-Michel Mesnard. Other antibodies used are as listed (supplemental Table S1).

### Adenovirus vectors

Recombinant adenovirus vectors Ad-Tax, Ad-tTA, Ad-RelA, Ad-RelB, and Ad-c-Rel were grown and titered as previously published [14].

### Derivation of HeLa cell lines

The cDNA for the degradation-resistant I-κBα, ΔN-I-κBα, was generated by PCR (see supplemental Table S2 for primer sequences). BamHI and EcoRI restriction endonuclease sites were used to subclone the cDNA into LV-SV-puro vector [18], [27]. HeLa/18×21-EGFP (HeLa-G) cells [18] were transduced with LV-ΔN-I-κBα-SV-puro, grown in DMEM containing fetal bovine serum (10%) and puromycin (1 µg/ml, *Sigma-Aldrich*) for one week with change of medium every 48 hours. HeLa-G/ΔN-I-κBα clones were then isolated in 96-well plates after limiting dilution and confirmed for ΔN-I-κBα expression by immunoblotting. The cDNA of the COOH-terminus Flag-tagged HBZ (Flag-HBZ) was generated by PCR (primer sequences listed in supplemental Table S2) using the cDNA of the spliced HBZ transcript as the template [45] and cloned into an MuLV-based retroviral vector, pBabe-Puro [30]. The pBabe-Flag-HBZ retroviral vector was produced as described [47]. HeLa-G transduced by the Flag-HBZ retroviral vector was selected in medium containing puromycin (1 µg/ml) and cloned by limiting dilution.

### Luciferase reporter assay

Each of the NF-κB, HTLV-1 LTR and p21 promoter constructs (E-selectin-Luc, LTR-Luc and p21-Luc) together with pRL-TK, a control plasmid that contains the HSV thymidine kinase (TK) promoter-driven *Renilla* luciferase reporter cassette (Promega), were used in the luciferase reporter assays according to standard procedures [17]. The p21 (−2.4 kb to +16)-Luc (also known as WWP-luc) was kindly provided by Dr. Wafik S EL-Deiry.

### Flow cytometry

Cells were harvested after trypsin treatment, and washed twice with 2 ml of cold phosphate-buffered saline solution (PBS), resuspended in 0.5 ml of PBS and fixed in 5 ml of 70% ethanol overnight at 4°C. Ethanol-fixed cells were then washed twice with 5 ml of cold PBS containing 1% bovine serum and incubated for 30 min at 37°C in 1.0 ml of a solution containing propidium iodide (PI) (20 µg/ml) and RNase (1 mg/ml), filtered through a 70-micron cell strainer (BD Falcon, Cat. 35-2350) to remove cell clumps. The cellular DNA content was determined by fluorescence-activated cell sorting (FACS) (EPICS XL-MCL flow cytometer; Beckman-Coulter). The percentages of cells in G0/G1, S, and G2/M phases of the cell cycle were computed using the ModFit LT software. Ordinate: cell numbers; abscissa: DNA content.

### Protein half-life determination and quantitation of p21 mRNA

The half-lives of p21 and p27 were measured as previously published [17]. The mRNA levels of p21 were measured by real-time PCR as described [17] using the primers listed (supplemental Table S2).

### Knockdown of NF-κB genes

HeLa-G cell lines with stable knockdown of each of the NF-κB members, RelA, RelB, c-Rel and p100, were isolated after screening cell clones (by immunoblotting) transduced by pooled shRNA lentiviral vectors containing the puromycin-resistance gene. The sequences targeted for each gene are as listed (supplemental Table S3).

### Accession numbers

The GENBANK accession numbers or ID numbers for genes encoding the proteins described in this article are: HTLV-1 Tax: AB038239; HTLV-1 HBZ: DQ273132; p21: NM_000389; p27: NM_004064; Cyclin B1: NM_031966; Skp2: NM_005983; I-κBα: NM_020529; p100/p52: NM_002502; p105/p50: NM_003998; RelA: NM_021975; RelB: NM_006509; and c-Rel: NM_002908.

## Supporting Information

Figure S1 (**A**) Ad-RelA activates NF-κB reporter. HeLa G cells were transfected with E-selectin-Luc and control *Renilla* luciferase plasmid, pRL-TK, as in Fig. 2A, and then transduced with increasing amounts (MOI: 0, 2.5, 10, 25, and 50) of Ad-RelA or Ad-tTa control. Luciferase activities and fold activation of the E-selectin-Luc reporter were determined as in Fig. 2A. (**B**) Over-expression of RelA, RelB or c-Rel does not cause senescence. HeLa G cells were transduced as in Fig. 3a with Ad-tTa, Ad-RelA, Ad-RelB and Ad-c-Rel vectors respectively at an MOI of 100 to ensure most of the cells were transduced. They were then monitored for 5 days and photographed.
(TIF)

Figure S2 NF-κB and LTR activation by Tax are attenuated by HBZ. (**A**) E-selectin-Luc and (**B**) LTR-Luc respectively were co-transfected with the control *Renilla* luciferase plasmid, pRL-TK, and increasing amounts of wild-type (WT) or null mutant (TTG) HBZ-expression plasmid as indicated. The total amount of DNA was kept constant by including the empty vector plasmid, pME-18Sneo when necessary. One day post-transfection, the medium was changed and the transfected cells were infected by Ad-Tax or Ad–tTa at an MOI of 5. Luciferase activities and fold trans-activation by Tax were calculated similarly as Fig. 2A. (**C**) Both untagged HBZ and Flag-epitope tagged HBZ inhibit NF-κB activation by Tax. HeLa G cells were co-transfected with E-selectin-Luc, pRL-TK, Tax-expression plasmid BC12-Tax, and increasing amounts of CMV-HBZ or CMV-HBZ-Flag plasmid. Luciferase activities were measured 48 hours after transfection and plotted as in (A) and (B).
(TIF)

Figure S3 Quantitative analysis of resistance to Tax-induced senescence. (**A**) HeLa-G and HeLa-G/ΔN-I-κBα L6 and H5 cells were plated sparsely (10,000 cells/well, approximately 1% confluency) on a 6-well plate. The cells were then transduced with the Ad-Tax vector. Five days after transduction, the degree of cellular senescence for each cell line was estimated by counting the foci of EGFP+ cells. The percentages of foci containing more than 4 EGFP+ cells were determined and plotted as an indicator of resistance to senescence. (**B**) HeLa-G (CTRL) and HeLa-G derivatives with RelA, RelB, c-Rel, and p100 knockdown (sh-RelA, sh-RelB, sh-c-Rel, and sh-p100) were transduced by Ad-Tax as in (A) and the degrees of resistance to Tax-induced senescence calculated and plotted. (**C**) Similar to (A) and (B) except the HeLa-G/Flag-HBZ cell line was analyzed.
(TIF)

Table S1 Antibodies used for immunoblots.
(RTF)

Table S2 Primers used for PCR.
(RTF)

Table S3 shRNA clones and their target sequences.
(RTF)

## References

[1] Taylor GP, Matsuoka M (2005). Natural history of adult T-cell leukemia/lymphoma and approaches to therapy.. Oncogene.

[2] Matsuoka M, Jeang KT (2007). Human T-cell leukaemia virus type 1 (HTLV-1) infectivity and cellular transformation.. Nat Rev Cancer.

[3] Siu YT, Chin KT, Siu KL, Yee Wai CE, Jeang KT (2006). TORC1 and TORC2 coactivators are required for tax activation of the human T-cell leukemia virus type 1 long terminal repeats.. J Virol.

[4] Harrod R, Kuo YL, Tang Y, Yao Y, Vassilev A (2000). p300 and p300/cAMP-responsive element-binding protein associated factor interact with human T-cell lymphotropic virus type-1 Tax in a multi-histone acetyltransferase/activator-enhancer complex.. J Biol Chem.

[5] Nyborg JK, Egan D, Sharma N (2009). The HTLV-1 Tax protein: Revealing mechanisms of transcriptional activation through histone acetylation and nucleosome disassembly.. Biochim Biophys Acta.

[6] Chu ZL, Shin YA, Yang JM, Di Donato JA, Ballard DWLH (1999). IKKgamma mediates the interaction of cellular IkappaB kinases with the tax transforming protein of human T cell leukemia virus type 1.. J Biol Chem.

[7] Jin DY, Giordano V, Kibler KV, Nakano H, Jeang KTLH (1999). Role of adapter function in oncoprotein-mediated activation of NF-kappaB. Human T-cell leukemia virus type I Tax interacts directly with IkappaB kinase gamma.. J Biol Chem.

[8] Xiao G, Sun SC (2000). Activation of IKKalpha and IKKbeta through their fusion with HTLV-I tax protein.. Oncogene.

[9] Grossman WJ, Kimata JT, Wong FH, Zutter M, Ley TJ (1995). Development of leukemia in mice transgenic for the tax gene of human T-cell leukemia virus type I.. Proc Natl Acad Sci U S A.

[10] Matsumoto K, Shibata H, Fujisawa JI, Inoue H, Hakura A (1997). Human T-cell leukemia virus type 1 Tax protein transforms rat fibroblasts via two distinct pathways.. J Virol.

[11] Yamaoka S, Inoue H, Sakurai M, Sugiyama T, Hazama M (1996). Constitutive activation of NF-kappa B is essential for transformation of rat fibroblasts by the human T-cell leukemia virus type I Tax protein.. EMBO J.

[12] Yamaoka S, Courtois G, Bessia C, Whiteside ST, Weil R (1998). Complementation cloning of NEMO, a component of the IkappaB kinase complex essential for NF-kappaB activation.. Cell.

[13] Hasegawa H, Sawa H, Lewis MJ, Orba Y, Sheehy N (2006). Thymus-derived leukemia-lymphoma in mice transgenic for the Tax gene of human T-lymphotropic virus type I.. Nat Med.

[14] Kuo YL, Giam CZ (2006). Activation of the anaphase promoting complex by HTLV-1 tax leads to senescence.. EMBO J.

[15] Carrano AC, Eytan E, Hershko A, Pagano M (1999). SKP2 is required for ubiquitin-mediated degradation of the CDK inhibitor p27.. Nat Cell Biol.

[16] Hara T, Kamura T, Nakayama K, Oshikawa K, Hatakeyama S (2001). Degradation of p27(Kip1) at the G(0)–G(1) transition mediated by a Skp2-independent ubiquitination pathway.. J Biol Chem.

[17] Zhang L, Zhi H, Liu M, Kuo YL, Giam CZ (2009). Induction of p21(CIP1/WAF1) expression by human T-lymphotropic virus type 1 Tax requires transcriptional activation and mRNA stabilization.. Retrovirology.

[18] Liu M, Yang L, Zhang L, Liu B, Merling R (2008). Human T-cell leukemia virus type 1 infection leads to arrest in the G1 phase of the cell cycle.. J Virol.

[19] Zhao T, Yasunaga J, Satou Y, Nakao M, Takahashi M (2009). Human T-cell leukemia virus type 1 bZIP factor selectively suppresses the classical pathway of NF-kappaB.. Blood.

[20] Gaudray G, Gachon F, Basbous J, Biard-Piechaczyk M, Devaux C (2002). The complementary strand of the human T-cell leukemia virus type 1 RNA genome encodes a bZIP transcription factor that down-regulates viral transcription.. J Virol.

[21] Lemasson I, Lewis MR, Polakowski N, Hivin P, Cavanagh MH (2007). Human T-cell leukemia virus type 1 (HTLV-1) bZIP protein interacts with the cellular transcription factor CREB to inhibit HTLV-1 transcription.. J Virol.

[22] Harrod R, Tang Y, Nicot C, Lu HS, Vassilev A (1998). An exposed kid-like domain in human t-cell lymphotropic virus type 1 tax is responsible for the recruitment of coactivators cbp/p300.. Mol Cell Biol.

[23] Smith MR, Greene WC (1990). Identification of HTLV-I tax trans-activator mutants exhibiting novel transcriptional phenotypes.. Genes Dev.

[24] Semmes OJ, Jeang KT (1992). Mutational analysis of human T-cell leukemia virus type I Tax: regions necessary for function determined with 47 mutant proteins.. J Virol.

[25] Brown K, Gerstberger S, Carlson L, Franzoso G, Siebenlist U (1995). Control of I kappa B-alpha proteolysis by site-specific, signal- induced phosphorylation.. Science.

[26] Brockman JA, Scherer DC, McKinsey TA, Hall SM, Qi X (1995). Coupling of a signal response domain in I kappa B alpha to multiple pathways for NF-kappa B activation.. Mol Cell Biol.

[27] Zhang L, Liu M, Merling R, Giam CZ (2006). Versatile reporter systems show that transactivation by human T-cell leukemia virus type 1 Tax occurs independently of chromatin remodeling factor BRG1.. J Virol.

[28] Sakaue-Sawano A, Kurokawa H, Morimura T, Hanyu A, Hama H (2008). Visualizing spatiotemporal dynamics of multicellular cell-cycle progression.. Cell.

[29] Basak S, Shih VF, Hoffmann A (2008). Generation and activation of multiple dimeric transcription factors within the NF-kappaB signaling system.. Mol Cell Biol.

[30] Morgenstern JP, Land H (1990). Advanced mammalian gene transfer: high titre retroviral vectors with multiple drug selection markers and a complementary helper- free packaging cell line.. Nucleic Acids Res.

[31] Merling R, Chen C, Hong S, Zhang L, Liu M (2007). HTLV-1 Tax mutants that do not induce G1 arrest are disabled in activating the anaphase promoting complex.. Retrovirology.

[32] Lin HK, Chen Z, Wang G, Nardella C, Lee SW (2010). Skp2 targeting suppresses tumorigenesis by Arf-p53-independent cellular senescence.. Nature.

[33] Cereseto A, Washington PR, Rivadeneira E, Franchini G (1999). Limiting amounts of p27Kip1 correlates with constitutive activation of cyclin E-CDK2 complex in HTLV-I-transformed T-cells.. Oncogene.

[34] Ping B, He X, Xia W, Lee DF, Wei Y (2006). Cytoplasmic expression of p21CIP1/WAF1 is correlated with IKKbeta overexpression in human breast cancers.. Int J Oncol.

[35] Adler AS, Kawahara TL, Segal E, Chang HY (2008). Reversal of aging by NFkappaB blockade.. Cell Cycle.

[36] Adler AS, Sinha S, Kawahara TL, Zhang JY, Segal E (2007). Motif module map reveals enforcement of aging by continual NF-kappaB activity.. Genes Dev.

[37] Bernard D, Gosselin K, Monte D, Vercamer C, Bouali F (2004). Involvement of Rel/nuclear factor-kappaB transcription factors in keratinocyte senescence.. Cancer Res.

[38] Bash J, Zong WX, Gelinas C (1997). c-Rel arrests the proliferation of HeLa cells and affects critical regulators of the G1/S-phase transition.. Mol Cell Biol.

[39] Seitz CS, Deng H, Hinata K, Lin Q, Khavari PA (2000). Nuclear factor kappaB subunits induce epithelial cell growth arrest.. Cancer Res.

[40] Adams PD (2009). Healing and hurting: molecular mechanisms, functions, and pathologies of cellular senescence.. Mol Cell.

[41] Landry S, Halin M, Vargas A, Lemasson I, Mesnard JM (2009). Upregulation of human T-cell leukemia virus type 1 antisense transcription by the viral tax protein.. J Virol.

[42] Arnold J, Yamamoto B, Li M, Phipps AJ, Younis I (2006). Enhancement of infectivity and persistence in vivo by HBZ, a natural antisense coded protein of HTLV-1.. Blood.

[43] Arnold J, Zimmerman B, Li M, Lairmore MD, Green PL (2008). Human T-cell leukemia virus type-1 antisense-encoded gene, Hbz, promotes T-lymphocyte proliferation.. Blood.

[44] Li M, Kesic M, Yin H, Yu L, Green PL (2009). Kinetic analysis of human T-cell leukemia virus type 1 gene expression in cell culture and infected animals.. J Virol.

[45] Satou Y, Yasunaga J, Yoshida M, Matsuoka M (2006). HTLV-I basic leucine zipper factor gene mRNA supports proliferation of adult T cell leukemia cells.. Proc Natl Acad Sci U S A.

[46] Saito M, Matsuzaki T, Satou Y, Yasunaga J, Saito K (2009). In vivo expression of the HBZ gene of HTLV-1 correlates with proviral load, inflammatory markers and disease severity in HTLV-1 associated myelopathy/tropical spastic paraparesis (HAM/TSP).. Retrovirology.

[47] Liang MH, Geisbert T, Yao Y, Hinrichs SH, Giam CZ (2002). Human T-lymphotropic virus type 1 oncoprotein tax promotes S-phase entry but blocks mitosis.. J Virol.

