# Supplementary figures and images for: NF-κB Hyper-Activation by HTLV-1 Tax Induces Cellular Senescence, but Can Be Alleviated by the Viral Anti-Sense Protein HBZ
Source: PLoS Pathog. 2011 Apr 28;7(4):e1002025. doi: 10.1371/journal.ppat.1002025 (PMC3084201; doi:10.1371/journal.ppat.1002025)

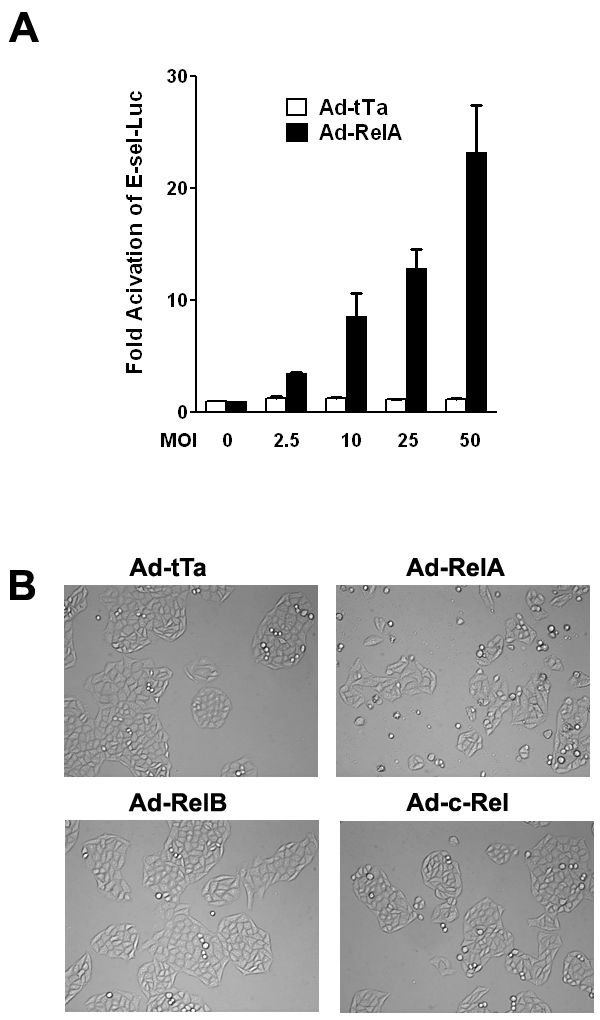

Supplement: Figure S1 — (A) Ad-RelA activates NF-κB reporter. HeLa G cells were transfected with E-selectin-Luc and control Renilla luciferase plasmid, pRL-TK, as in Fig. 2A, and then transduced with increasing amounts (MOI: 0, 2.5, 10, 25, and 50) of Ad-RelA or Ad-tTa control. Luciferase activities and fold activation of the E-selectin-Luc reporter were determined as in Fig. 2A. (B) Over-expression of RelA, RelB or c-Rel does not cause senescence. HeLa G cells were transduced as in Fig. 3a with Ad-tTa, Ad-RelA, Ad-RelB and Ad-c-Rel vectors respectively at an MOI of 100 to ensure most of the cells were transduced. They were then monitored for 5 days and photographed. (TIF) [file ppat.1002025.s001.tif]

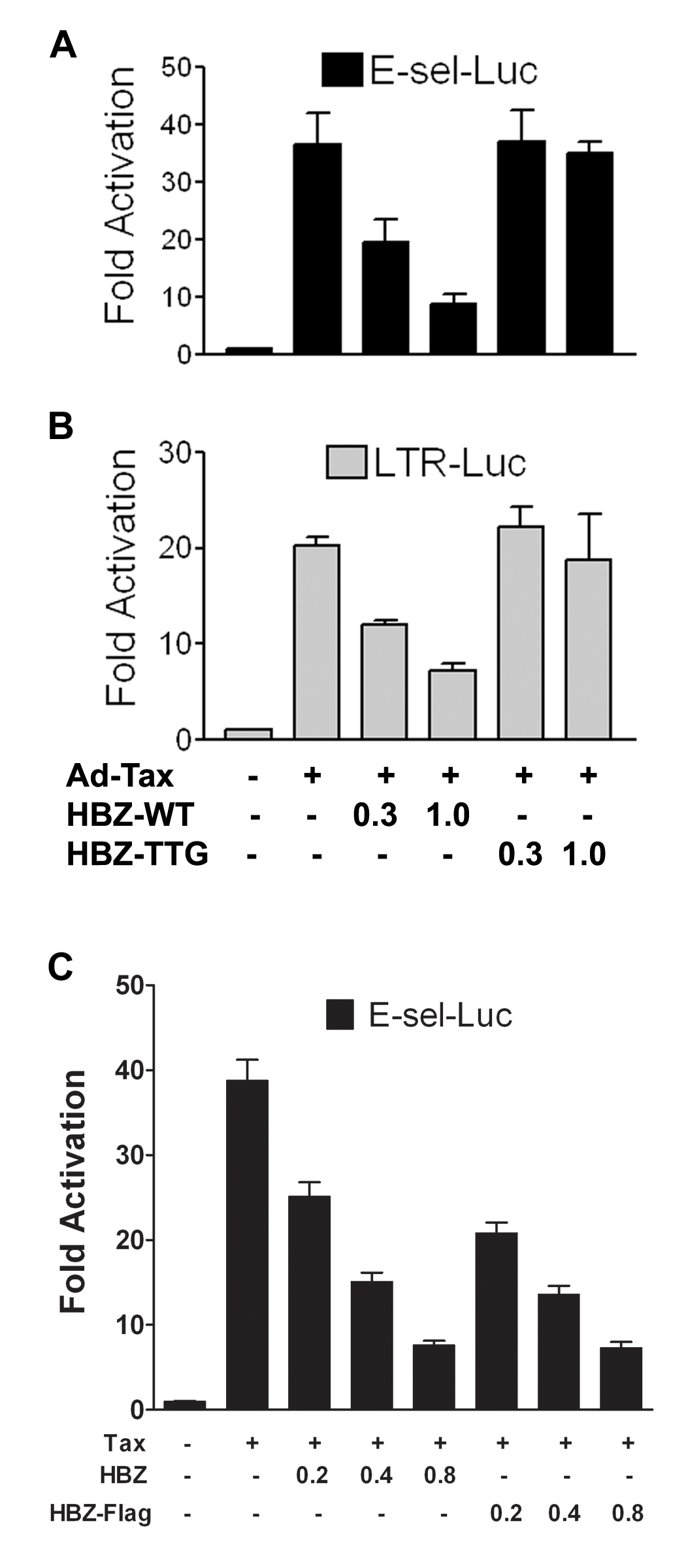

Supplement: Figure S2 — NF-κB and LTR activation by Tax are attenuated by HBZ. (A) E-selectin-Luc and (B) LTR-Luc respectively were co-transfected with the control Renilla luciferase plasmid, pRL-TK, and increasing amounts of wild-type (WT) or null mutant (TTG) HBZ-expression plasmid as indicated. The total amount of DNA was kept constant by including the empty vector plasmid, pME-18Sneo when necessary. One day post-transfection, the medium was changed and the transfected cells were infected by Ad-Tax or Ad–tTa at an MOI of 5. Luciferase activities and fold trans-activation by Tax were calculated similarly as Fig. 2A. (C) Both untagged HBZ and Flag-epitope tagged HBZ inhibit NF-κB activation by Tax. HeLa G cells were co-transfected with E-selectin-Luc, pRL-TK, Tax-expression plasmid BC12-Tax, and increasing amounts of CMV-HBZ or CMV-HBZ-Flag plasmid. Luciferase activities were measured 48 hours after transfection and plotted as in (A) and (B). (TIF) [file ppat.1002025.s002.tif]

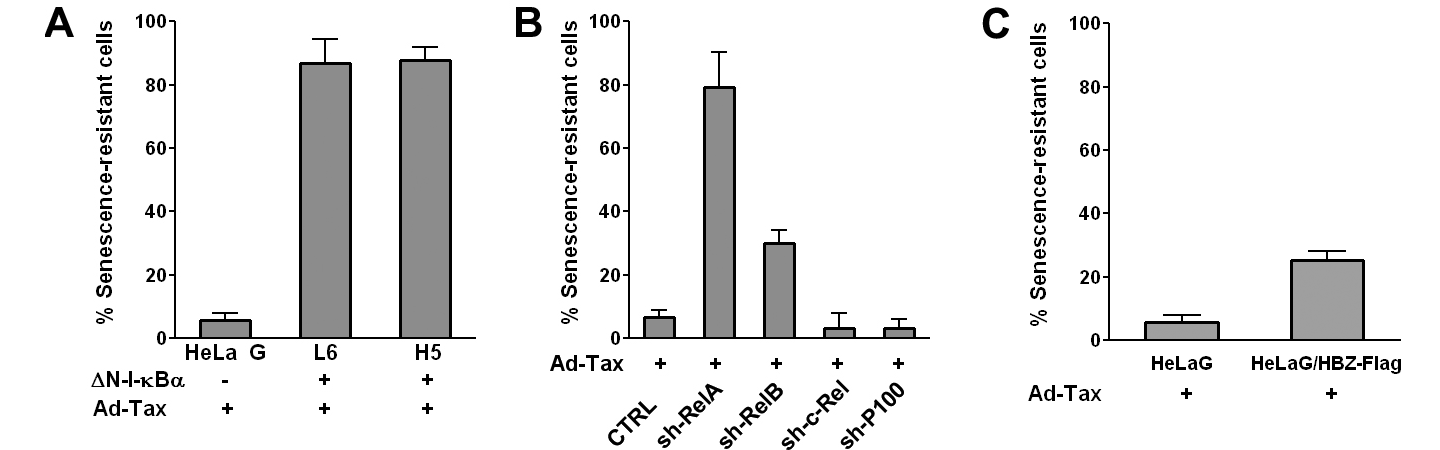

Supplement: Figure S3 — Quantitative analysis of resistance to Tax-induced senescence. (A) HeLa-G and HeLa-G/ΔN-I-κBα L6 and H5 cells were plated sparsely (10,000 cells/well, approximately 1% confluency) on a 6-well plate. The cells were then transduced with the Ad-Tax vector. Five days after transduction, the degree of cellular senescence for each cell line was estimated by counting the foci of EGFP+ cells. The percentages of foci containing more than 4 EGFP+ cells were determined and plotted as an indicator of resistance to senescence. (B) HeLa-G (CTRL) and HeLa-G derivatives with RelA, RelB, c-Rel, and p100 knockdown (sh-RelA, sh-RelB, sh-c-Rel, and sh-p100) were transduced by Ad-Tax as in (A) and the degrees of resistance to Tax-induced senescence calculated and plotted. (C) Similar to (A) and (B) except the HeLa-G/Flag-HBZ cell line was analyzed. (TIF) [file ppat.1002025.s003.tif]
