# Supplementary material for: NF-κB Hyper-Activation by HTLV-1 Tax Induces Cellular Senescence, but Can Be Alleviated by the Viral Anti-Sense Protein HBZ
Source: PLoS Pathog. 2011 Apr 28;7(4):e1002025. doi: 10.1371/journal.ppat.1002025 (PMC3084201; doi:10.1371/journal.ppat.1002025)
Supplement: Table S1 — Antibodies used for immunoblots. (RTF) [file ppat.1002025.s004.rtf]

Supplemental Table 1: Antibodies used for immunoblots.

Antibody	Catalog number	Vender	
Cyclin B1	SC-752	Santa Cruz Biotechnology	
p21CIP1/WAF1	SC-397	Santa Cruz Biotechnology	
p27KIP1	SC-1641	Santa Cruz Biotechnology	
I-kBa	SC-847	Santa Cruz Biotechnology	
RelA	SC-8008	Santa Cruz Biotechnology	
RelB	SC-48379	Santa Cruz Biotechnology	
p100/p52	SC-7386	Santa Cruz Biotechnology	
b-actin	SC-1616	Santa Cruz Biotechnology	
c-Rel	4727	Cell Signaling Technology	
p105/p50	3035	Cell Signaling Technology	
Skp2	32–3400	Zymed Laboratories	


 
