# Supplementary material for: NF-κB Hyper-Activation by HTLV-1 Tax Induces Cellular Senescence, but Can Be Alleviated by the Viral Anti-Sense Protein HBZ
Source: PLoS Pathog. 2011 Apr 28;7(4):e1002025. doi: 10.1371/journal.ppat.1002025 (PMC3084201; doi:10.1371/journal.ppat.1002025)
Supplement: Table S2 — Primers used for PCR. (RTF) [file ppat.1002025.s005.rtf]

Supplemental Table 2: Primers used for PCR.

Primer	Sequence	
N-I-kBa-F	5'-CCTGGATCCGCCACCATGAAAGACGAGGAGT	
N-I-kBa-R	5'- GGCGAATTCATAACGTCAGACGCT	
Flag-HBZ-F                       	5'-TAGGATCCGCCCACCATGGCGGCCTCAGGGCTG	
Flag-HBZ-R	5'- ATGAATTCTTAGTACTTATCGTCGTCATCCTTGTAATCT  TGCAACCACATCGCCTCCAGCCTC 	
P21-F	5'-CCATGTGGACCTGTCACTGT	
P21-R	5'-TGGTAGAAATCTGTCATGCTGGTC	
18S rRNA-F	5'-CGGCTACCACATCCAAGGAA	
18S rRNA-R	5'- GCTGGAATTACCGCGGCT	

 
