# Supplementary material for: NF-κB Hyper-Activation by HTLV-1 Tax Induces Cellular Senescence, but Can Be Alleviated by the Viral Anti-Sense Protein HBZ
Source: PLoS Pathog. 2011 Apr 28;7(4):e1002025. doi: 10.1371/journal.ppat.1002025 (PMC3084201; doi:10.1371/journal.ppat.1002025)
Supplement: Table S3 — shRNA clones and their target sequences. (RTF) [file ppat.1002025.s006.rtf]

Supplemental Table 3: shRNA clones and their target sequences

Gene symbol	Clone ID	NM ID	Target sequence	
RelA	TRCN14683	NM_021975	GCCTTAATAGTAGGGTAAGTT	
RelA	TRCN14684	NM_021975	CGGATTGAGGAGAAACGTAAA	
RelA	TRCN14685	NM_021975	GCAGGCTATCAGTCAGCGCAT	
RelA	TRCN14686	NM_021975	CACCATCAACTATGATGAGTT	
RelA	TRCN14687	NM_021975	CCTGAGGCTATAACTCGCCTA	
RelB	TRCN14713	NM_006509	CATGCTTCTGAAGTGGACATA	
RelB	TRCN14714	NM_006509	GCTGCGGATTTGCCGAATTAA	
RelB	TRCN14715	NM_006509	CACAGATGAATTGGAGATCAT	
RelB	TRCN14716	NM_006509	AGCCCGTCTATGACAAGAAAT	
RelB	TRCN14717	NM_006509	CGAGAGCAAACGGCGGAAGAA	
c-Rel	TRCN39983	NM_002908	CCACCTATATAGATGCAGCAT	
c-Rel	TRCN39984	NM_002908	GCAGGAATCAATCCATTCAAT	
c-Rel	TRCN39985	NM_002908	GCTGTCTAATTGTTCTGTGAA	
c-Rel	TRCN39986	NM_002908	CCAGGAAGTTAGTGAATCTAT	
c-Rel	TRCN39987	NM_002908	GCAGATAACAGCATGATAAAT	
c-Rel	TRCN10420	NM_002908	GAAGATTGTGACCTCAATGTG	
c-Rel	TRCN10421	NM_002908	CTTCAGTTGTGCAGATAACAG	
NFkB2(p100/p52)	TRCN06512	NM_002502	GCTGCTAAATGCTGCTCAGAA	
NFkB2(p100/p52)	TRCN06513	NM_002502	CGATTTCGATATGGCTGTGAA	
NFkB2(p100/p52)	TRCN06514	NM_002502	CCCTATCACAAGATGAAGATT	
NFkB2(p100/p52)	TRCN06515	NM_002502	CCTGTAACAGTGTTTCTGCAA	
NFkB2(p100/p52)	TRCN06516	NM_002502	GCCCAATTTAACAACCTGGGT	


 
